# A patient with recurrent palpitations and unusual anatomy

**DOI:** 10.1007/s12471-020-01404-2

**Published:** 2020-03-19

**Authors:** C. J. M. Lawson, A. D. Margulescu, J. Barry

**Affiliations:** Department of Cardiology, Morriston Regional Cardiac Centre, SA6 6NL Swansea, UK

A 49-year-old woman was admitted for an electrophysiology study and ablation of recurrent episodes of symptomatic narrow complex tachycardia. Through a right inguinal vascular approach, a deflectable decapolar and a non-deflectable quadripolar electrophysiology catheter were placed into the cardiac chambers following the trajectory depicted in Fig. 1. In addition, using a right subclavian venous approach, a 4 mm non-irrigated tip ablation catheter (Celsius, Biosense Webster, USA) was used to map the triangle of Koch.

**Fig. 1:**
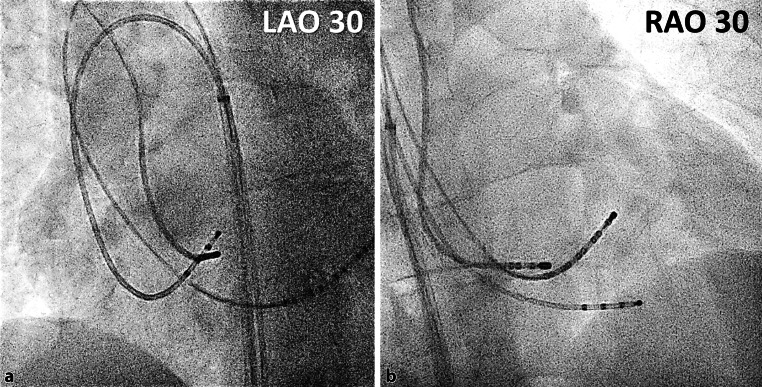
The position of the electrophysiology catheters depicting the peculiar route through which the 2 electrophysiology catheters from the right inguinal region were placed inside the heart.** a** left anterior oblique 30 degrees view, LAO 30; **b** right anterior oblique 30 degrees view, RAO 30

Typical atrioventricular nodal re-entrant tachycardia (AVNRT) was diagnosed at the electrophysiology study, and a slow pathway (SP) ablation was successfully performed at the right posteroseptal region in the usual fashion. There were no complications, and no recurrences of the tachycardia at follow-ups.

What is the vascular abnormality that accounts for the peculiar route through which the 2 electrophysiology catheters from the right inguinal region were placed in the heart?

## Answer

You will find the answer elsewhere in this issue.

